# Enhanced membrane protein production in HEK293T cells via *ATF4* gene knockout: A CRISPR-Cas9 mediated approach

**DOI:** 10.17305/bb.2024.11519

**Published:** 2025-01-21

**Authors:** Byung-Jo Choi, Ba Reum Kim, Ho Joong Choi, Ok-Hee Kim, Say-June Kim

**Affiliations:** 1 Department of Surgery, Daejeon St. Mary’s Hospital, College of Medicine, The Catholic University of Korea, Seoul, Republic of Korea; 2 Catholic Central Laboratory of Surgery, College of Medicine, The Catholic University of Korea, Seoul, Republic of Korea; 3 Translational Research Team, Surginex Co., Republic of Korea; 4 Department of Surgery, Seoul St. Mary’s Hospital, College of Medicine, The Catholic University of Korea, Seoul, Republic of Korea

**Keywords:** CRISPR-Cas9, gene editing, HEK293T cells, *ATF4* knockout, KO, membrane protein production

## Abstract

HEK293T cells are extensively utilized for therapeutic protein production due to their human origin, which enables accurate post-translational modifications. This study aimed to enhance membrane protein production in HEK293T cells by knocking out the *ATF4* gene using CRISPR-Cas9 technology. The *ATF4* gene was edited by infecting HEK293T cells with a lentivirus carrying optimized single-guide RNA (ATF4-KO-3) and Cas9 genes. Comparative evaluations were conducted using all-in-one and two-vector systems. Genome sequencing and membrane protein productivity of ATF4-knockout (KO) cells were compared to wild-type (WT) cells using next-generation sequencing (NGS) and a membrane protein isolation kit, respectively. Single-cell analysis confirmed gene editing patterns, with NGS verifying the intended deletions. Membrane protein production was also assessed indirectly via flow cytometry, analyzing cells expressing Membrane-GFP. Compared to WT cells, ATF4-KO cells exhibited a significant increase in membrane protein production, with a 52.2 ± 19.0% improvement. Gene editing efficiency was compared between the two delivery systems, with the two-vector system demonstrating higher efficiency based on T7 endonuclease I assays. Western blot analysis confirmed *ATF4* suppression and increased expression of membrane proteins, including E-cadherin and CD63. Quantitative analysis via PAGE revealed a 77.2 ± 30.6% increase in purified membrane protein yields, consistent with the observed enhancements. Flow cytometry using Membrane-GFP further demonstrated a 22.9 ± 9.7% increase in productivity. In summary, *ATF4* knockout significantly enhances membrane protein production in HEK293T cells, offering potential improvements in biopharmaceutical manufacturing by enabling more efficient protein synthesis.

## Introduction

HEK293T cells are widely used in the medical industry for producing recombinant proteins and viral vectors. Their human origin makes them particularly advantageous for biologics intended for human use, as they ensure accurate post-translational modifications and reduce immunogenicity compared to non-human cell lines like CHO cells [1–3]. These attributes make HEK293T cells ideal for manufacturing therapeutic proteins and viral vectors used in gene therapy, vaccine development, and cancer treatments. To enhance protein production in HEK293T cells, various strategies have been employed, such as transient transfection, stable cell line development, and site-specific integration via recombinase-mediated cassette exchange [4]. Although these approaches have improved yields, challenges remain, including scalability, reproducibility, and the complexity of achieving stable, high-yield production. Furthermore, optimizing culture conditions, media composition, and bioprocess parameters is critical for maximizing production efficiency [5, 6]. *ATF4* is a transcription factor central to the integrated stress response and the unfolded protein response. During endoplasmic reticulum (ER) stress, Protein kinase RNA-like ER kinase (PERK) phosphorylates eIF2α, leading to increased ATF4 translation. Depending on the context and its binding partners, *ATF4* can promote either cell survival or apoptosis [7, 8]. Previous studies suggest that knocking out *ATF4* may reduce ER stress-induced apoptosis, potentially allowing cells to allocate more resources to protein synthesis and processing [9]. This hypothesis forms the basis for investigating whether *ATF4* knockout (KO) could enhance membrane protein production in HEK293T cells [5, 10]. This study aims to evaluate the effects of *ATF4* KO on membrane protein production in HEK293T cells using the CRISPR-Cas9 system. By targeting the *ATF4* gene, we hypothesize that reducing ER stress will improve cell survival and enhance protein production capacity. This research could offer valuable insights into optimizing biopharmaceutical production processes and developing more efficient cell lines for therapeutic protein manufacturing.

**Table 1:** Primer list for the study

**Primer name**	**Description**	**Primer sequences**
ATF4-KO-1_F^a^	Primer for CRISPR/Cas9 construction (ATF4-KO-1)	CACCGTACACCTTGGCTGTTG TTGG
ATF4-KO-1_R^a^		AAACCACCAACAACAGCAAGG GTGAC
ATF4-KO-2_F^a^	Primer for CRISPR/Cas9 construction (ATF4-KO-2)	CACCGCACTCACCTTGCTGTT GTTGT
ATF4-KO-2_R^a^		AAACACAACAACAGCAAGGGT GAGTGC
ATF4-KO-3_F^a^	Primer for CRISPR/Cas9 construction (ATF4-KO-3)	CACCGGTCCCTCCAAACAACA GCAA
ATF4-KO-3_R^a^		AAACTTGCTGTTGTTGGAGGG ACC
ATF4-T7E1_F^b^	Primer for T7 endonuclease I assay (Product size – 1,182 bp)	GGCGCGGGTTTTGGATTGGT
ATF4-T7E1_R^b^		TGGCCAATTGGGTTCACCGT
ATF4-NGS_F^c^	Primer for NGS analysis (Product size – 333 bp)	TCCTGAGCAGCGAGGTGTTGG T
ATF4-NGS_R^c^		TCTGTCCCGGAGAAGGCATCC TCTG

This is a list of primers used to create CRISPR/Cas9 constructs for *ATF4* genes and to analyze the results of *ATF4* genes. ^a^For cloning, Forward and Reverse primers were annealed together, and then ligation was performed using T4 ligase, ^b^These are T7E1 primers for PCR amplification of the target region of *ATF4* gene used in the T7E1 assay, and ^c^Primers for NGS for edited sequence analysis of *ATF4*. NGS: Next-generation sequencing; KO: Knockout.

## Materials and methods

### Cell culture

HEK 293T cells were obtained from the Korean Cell Line Bank (Seoul, Republic of Korea). The cells were cultured in Dulbecco’s Modified Eagle’s Medium (DMEM; Hyclone, Logan, UT), supplemented with 10% fetal bovine serum (FBS; Hyclone, Logan, UT, USA), 50 units/mL penicillin, and 50 µg/mL streptomycin (GibcoBRL, Carlsbad, CA). Cultures were maintained at 37 ^∘^C in a humidified atmosphere containing 5% CO_2_.

### Construction of plasmid DNA vectors

The lentiviral vector pL-CRISPR.EFS.tRFP (kindly provided by Benjamin Ebert; Addgene plasmid #57819, http://n2t.net/addgene: 57819, RRID) was used as the starting material. To create a single guide RNA (sgRNA) expression vector, designated pL-sgRNA.EFS.Zeo.tRFP, the Cas9 gene in the original vector was replaced with a bleomycin resistance gene. Additionally, a separate lentiviral vector, pLV-Cas9.SFFV.Neo, which co-expresses the Cas9 protein and a neomycin resistance gene, was obtained from VectorBuilder (Guangzhou, China). Finally, a lentiviral vector enabling the stable expression of a membrane-bound form of green fluorescent protein (mGFP) was engineered by cloning the GAP43 palmitoylation signal peptide sequence along with GFP into the pLVX-CMV-IRES-Puro vector (manufactured by Takara Bio, Inc., Otsu, Japan).

### Designing and production of sgRNA targeting *ATF4*

Utilizing the CRISPR RGEN Tool’s Cas-Designer (http://www.rgenome.net/cas-designer/), sgRNAs targeting the ATF4 gene were designed, resulting in the creation of three sgRNAs: ATF4-KO-1, ATF4-KO-2, and ATF4-KO-3 (Table 1). To develop sgRNA vectors suitable for screening, oligonucleotide pairs encoding the ATF4-specific sgRNA sequences were synthesized by Bionics (Seoul, Republic of Korea). These oligonucleotide pairs were annealed and cloned into the pL-CRISPR.EFS.tRFP and pL-sgRNA.EFS.Zeo.tRFP vectors to generate the corresponding sgRNA expression constructs. Consequently, three distinct sgRNA vectors—ATF4-KO-1, ATF4-KO-2, and ATF4-KO-3—were constructed. These vectors were then used to evaluate their efficiency in achieving optimal KO of the ATF4 gene through CRISPR-Cas9-mediated genome editing.

### T7 endonuclease I (T7E1) assay

To evaluate sgRNA activity, HEK293T cells were transfected with the pL-CRISPR.EFS.tRFP vector containing each sgRNA (ATF4-KO-1 to ATF4-KO-3) using Lipofectamine 2000 (Thermo Fisher Scientific, Waltham, MA, USA). Three days post-transfection, 5 × 10^6^ cells were harvested, and genomic DNA from each sample (wild-type [WT], ATF4-KO-1, ATF4-KO-2, and ATF4-KO-3) was extracted using the G-DEX IIc Genomic DNA Extraction Kit (iNtRON Biotechnology, Seoul, Republic of Korea), following the manufacturer’s protocol. To amplify the ATF4 target region, PCR was performed using the EzPC HF 5× PCR Master Mix (Elpis Biotech, Daejeon, Republic of Korea) and a specific primer pair (Table 1). The PCR protocol consisted of two steps: First step: one cycle at 95 ^∘^C for 5 min, followed by 10 cycles of 95 ^∘^C for 30 s, 70 ^∘^C for 30 s (with a 1 ^∘^C decrease per cycle to 60 ^∘^C), and 72 ^∘^C for 2 min. Second step: 30 cycles of 95 ^∘^C for 30 s, 60 ^∘^C for 30 s, 72 ^∘^C for 2 min, and a final extension at 72 ^∘^C for 3 min. Following amplification, a hybrid DNA duplex was formed using the following cycling method: 95 ^∘^C for 2 min, ramping down at −2^∘^C/s from 95 ^∘^C to 85 ^∘^C, and at −0.1^∘^C/s from 85 ^∘^C to 25 ^∘^C. For the T7E1 assay, a 20 µL reaction mixture (containing 2 µL of 10× NEB buffer-2, 10 units of T7E1 enzyme, and 10 µL of hybrid DNA duplex) was incubated at 37 ^∘^C for 20 min. The T7E1 digestion products were separated on a 1.5% agarose gel in TAE buffer containing 1× RedSafe Nucleic Acid Staining Solution (iNtRON Biotechnology) by electrophoresis and visualized under Blue LED light.

### Generation of stable HEK293T cell Lines with Cas9, mGFP, and targeted sgRNAs

Lentiviral vectors were used to establish HEK293T cell lines stably expressing Cas9 or mGFP. For this purpose, lentiviruses containing either the pLV-Cas9.SFFV.Neo or pLVX-CMV.mGFP.IRES. Puro vectors were transduced into HEK293T cells. Stable Cas9-expressing cell lines were selected using G418 (1000 µg/mL; InvivoGen, San Diego, CA, USA), whereas stable mGFP-expressing cell lines were selected with Puromycin (2 µg/mL; InvivoGen) over a two-weekperiod. Lentiviral vectors were also employed to generate genome-edited cell lines with ATF4 gene KO using CRISPR-Cas9 technology. Briefly, HEK293T cells were transduced with lentiviruses carrying pL-sgRNA.EFS.Zeo.tRFP vectors, each encoding a designed sgRNA targeting specific genomic loci. After transduction, cells infected with the sgRNA-expressing lentiviruses underwent rigorous selection using Zeocin (200 µg/mL; InvivoGen) for two weeks to ensure that only successfully transduced cells survived and proliferated. To create single-cell clones of ATF4-KO cells, the selected ATF4-KO stable cells were seeded into 96-well plates at a density of two cells per well using the limiting dilution method. After two weeks of incubation, wells containing individual clones were identified. Genomic DNA was extracted from these clones, and the ATF4 target region was amplified via PCR. The PCR products were analyzed using the T7E1 assay to identify clones with high editing efficiency. Four selected clones were further analyzed by next-generation sequencing (NGS) to assess gene-editing efficiency and mutation patterns.

### NGS

Five million (5 × 10^6^) ATF4-KO-HEK293T cells were harvested, and genomic DNA was purified from each sample (WT and ATF4-KO-3) using the Genomic DNA Extraction Kit, following the manufacturer’s instructions. To amplify the ATF4 gene region, PCR was performed using a specifically designed primer pair (Table 1). Each 40 µL PCR reaction contained 8 µL of 5× PCR Master Mix, 1 µL of each primer (10 pmol/µL), 2 µL of genomic DNA, and 28 µL of distilled water. The PCR protocol consisted of two steps. The first step began with initial denaturation at 95 ^∘^C for 3 min, followed by 10 cycles of denaturation at 95 ^∘^C for 10 s, annealing at 70 ^∘^C (decreasing by 1 ^∘^C per cycle to 60 ^∘^C) for 10 s, and extension at 72 ^∘^C for 30 s. In the second step, 30 cycles were performed with denaturation at 95 ^∘^C for 10 s, annealing at 60 ^∘^C for 10 s, and extension at 72 ^∘^C for 30 s, followed by a final extension at 72 ^∘^C for 3 min. PCR products were separated on a 1.5% agarose gel in TAE buffer containing 1× RedSafe Nucleic Acid Staining Solution (iNtRon Biotechnology) via electrophoresis and visualized under Blue LED light. Bands matching the expected size were excised from the gel, and the PCR products were extracted using the Labopass Gel Extraction Kit (Cosmogenetech, Seoul, Republic of Korea) in accordance with the manufacturer’s protocol. The purified PCR products were then sent for analysis by an NGS service provider (Bionics, Seoul, Republic of Korea).

### Determination of membranous protein yields

For the extraction of membrane proteins, 5 × 10^6^ cells from both ATF4-KO and WT HEK293T cell lines were collected. Membrane protein extraction was performed using the Mem-PER™ Plus Membrane Protein Extraction Kit (Thermo Fisher Scientific, Inc.) according to the manufacturer’s protocol. The harvested cells were washed twice with Cell Wash Solution and permeabilized in 0.75 mL of Permeabilization Buffer, followed by incubation at 4 ^∘^C for 10 min with shaking. After incubation, the samples were centrifuged at 16,000 × *g* for 15 min at 4 ^∘^C to separate the supernatant from the pellet. The supernatant was discarded, and the pellet was resuspended in 0.5 mL of Solubilization Buffer. The resuspended samples were incubated for 30 min at 4 ^∘^C with shaking. Following incubation, the samples were centrifuged again at 16,000 × *g* for 15 min at 4 ^∘^C to separate the supernatant and pellet. The membrane protein fraction (supernatant) was then collected. The Bradford assay was used to quantify the yield of extracted membrane proteins. For the assay, 20 µL of the extracted membrane proteins were diluted in 180 µL of distilled water, and 10 µL of the diluted sample was mixed with 200 µL of 1× Bradford reagent (Bio-Rad Protein Assay Dye Reagent Concentrate; Bio-Rad, Hercules, CA). After a 5-min incubation, absorbance was measured at 595 nm using a photometer (SpectraMax^®^ ABS; Molecular Devices, San Jose, CA, USA).

### Western blot analysis

To analyze protein expression in ATF4-KO-3 stable HEK293T cells, both WT and ATF4-KO cells were collected, and whole-cell lysates were extracted using RIPA lysis buffer. The purified lysates were analyzed by western blot using antibodies targeting various membrane proteins (E-cadherin, N-cadherin, and CD63), *ATF4*, and loading controls (α-tubulin and β-catenin). For qualitative analysis of membrane proteins, the protein samples were separated on a 12% polyacrylamide gel and transferred onto nitrocellulose membranes (Amersham Pharmacia Biotech Inc., Piscataway, NJ, USA). The following primary antibodies were used: E-cadherin (Cell Signaling Technology, Boston, MA, USA), N-cadherin (Cell Signaling Technology), CD63 (Cell Signaling Technology), α-tubulin (Santa Cruz Biotechnology, Santa Cruz, CA, USA), and β-catenin (Cell Signaling Technology). After protein transfer, the membranes were incubated with peroxidase-conjugated secondary antibodies, and the chemiluminescence signals of the target proteins were detected using an enhanced chemiluminescence system with a CCD camera imaging system (Atto, Tokyo, Japan).

### Flow cytometric analysis

To measure the mGFP intensity of ATF4-KO mGFP-stable HEK293T cells, ATF4-KO and WT mGFP-stable HEK293T cells were seeded into 100 mm cell culture dishes prior to the experiment. The cells were cultured to approximately 100% confluency and then harvested by trypsinization. The harvested cells were washed twice with PBS and subsequently resuspended in PBS. The resuspended cells were transferred to new FACS tubes, and GFP intensity was measured using the Attune NxT flow cytometer (Thermo Fisher, Inc.).

### Statistical analysis

Data are presented as means ± SEM. Statistical comparisons between two groups were made using an unpaired *t*-test, with significance set at *P* < 0.05. Analyses were based on three independent experiments.

## Results

### Optimizing sgRNA efficiency for ATF4-KO

**Figure 1.**
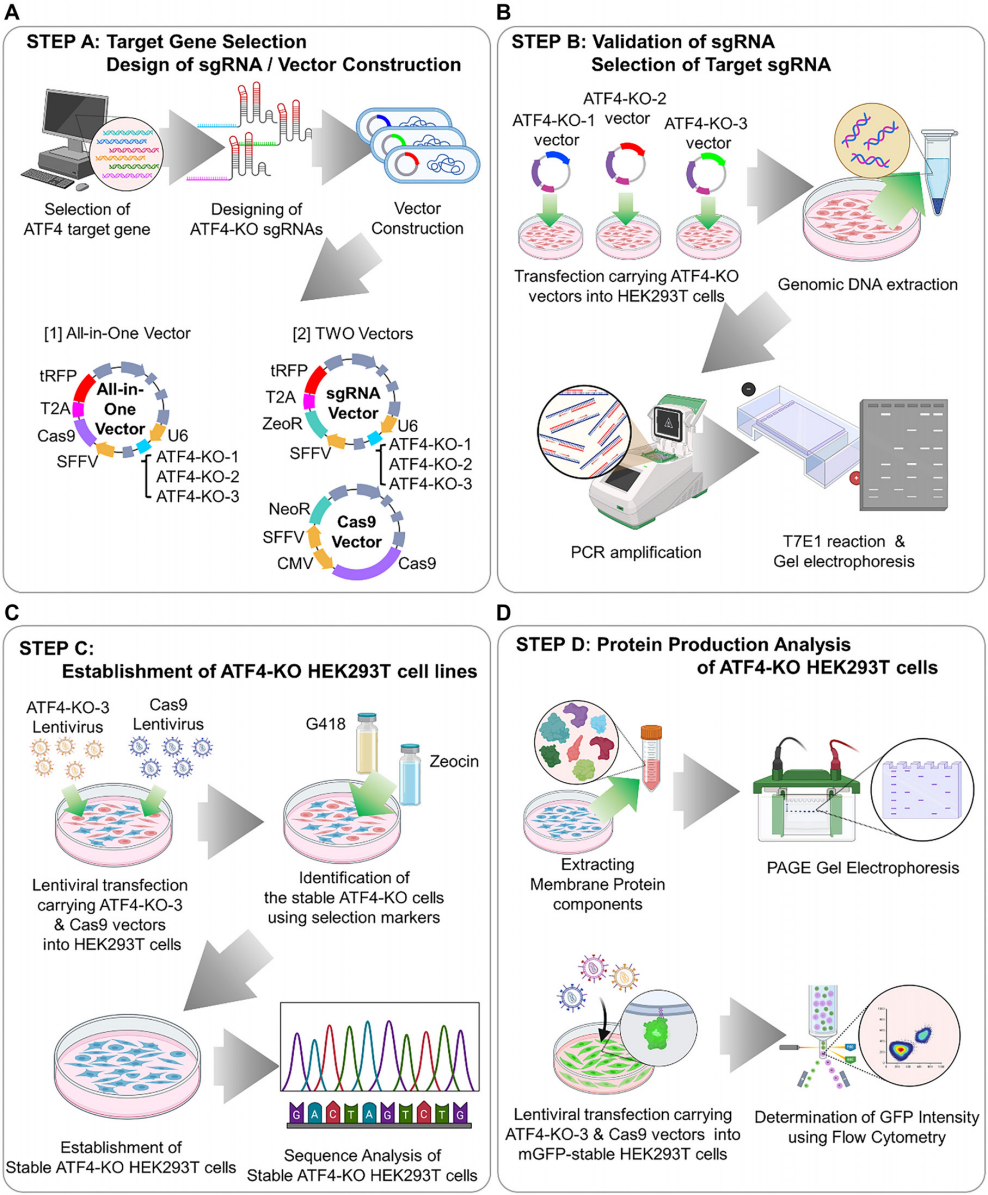
**Schematic diagram of developing a cell line with increased membrane protein productivity through *ATF4* gene editing.** (A) Step A: Gene selection and sgRNA design. *ATF4* was identified as a target for enhancing membrane protein productivity through a literature review. Three sgRNAs targeting *ATF4* were designed and cloned into both all-in-one and two-vector systems. (B) Step B: Validation of sgRNA and selection of target sgRNA. HEK293T cells were transfected with each sgRNA in an all-in-one vector. Genomic DNA was purified, and the target gene was amplified via PCR. The T7E1 assay identified ATF4-KO-3 as the sgRNA with the highest gene correction efficiency. (C) Step C: Lentiviral delivery and selection. The selected ATF4-KO-3 sgRNA and Cas9 were packaged into lentivirus particles and transduced into HEK293T cells. Cells were selected using G418 and Zeocin, and mutations were confirmed through genetic analysis. (D) Step D: Protein purification and analysis. Membrane proteins from the modified cell lines were purified, and yields were compared using SDS-PAGE and flowcytometry to confirm expression level. KO: Knockout; sgRNA: Single guide RNA; mGFP: Membrane-bound form of green fluorescent protein.

The experimental procedure to increase membrane protein production is outlined below (Figure 1). First, a literature review was conducted to identify the candidate *ATF4* gene and to design sgRNAs targeting it. To optimize *ATF4* gene editing, we planned experiments to suppress *ATF4* expression, thereby inducing the activation of downstream gene expression. This approach was informed by strategies from previous studies [11, 12]. Two types of vectors were constructed to suit experimental needs: an all-in-one vector, which expresses both Cas9 and sgRNA within a single vector, and a Two-vector system, which expresses Cas9 and sgRNA separately. In the second step, the constructed sgRNAs were introduced into HEK293T cells, and their efficiency was evaluated using the T7E1 assay. The sgRNA with the highest efficiency was selected for further experiments. In the third step, the selected sgRNA and Cas9 were packaged into lentiviral particles and used to infect HEK293T cells. Stable expression of the gene-editing components was ensured by selecting cells with Zeocin (for sgRNA) and G418 (for Cas9). Sequencing was performed to confirm the genetic editing patterns in these selected cells. Finally, membrane proteins were purified from both the stable cell line and the WT cell line to compare production levels. Additionally, the impact of *ATF4* editing on membrane protein production was indirectly assessed using flow cytometry by analyzing cells expressing Membrane-GFP.

### Validation of sgRNA efficiency using the T7E1 assay

**Figure 2.**
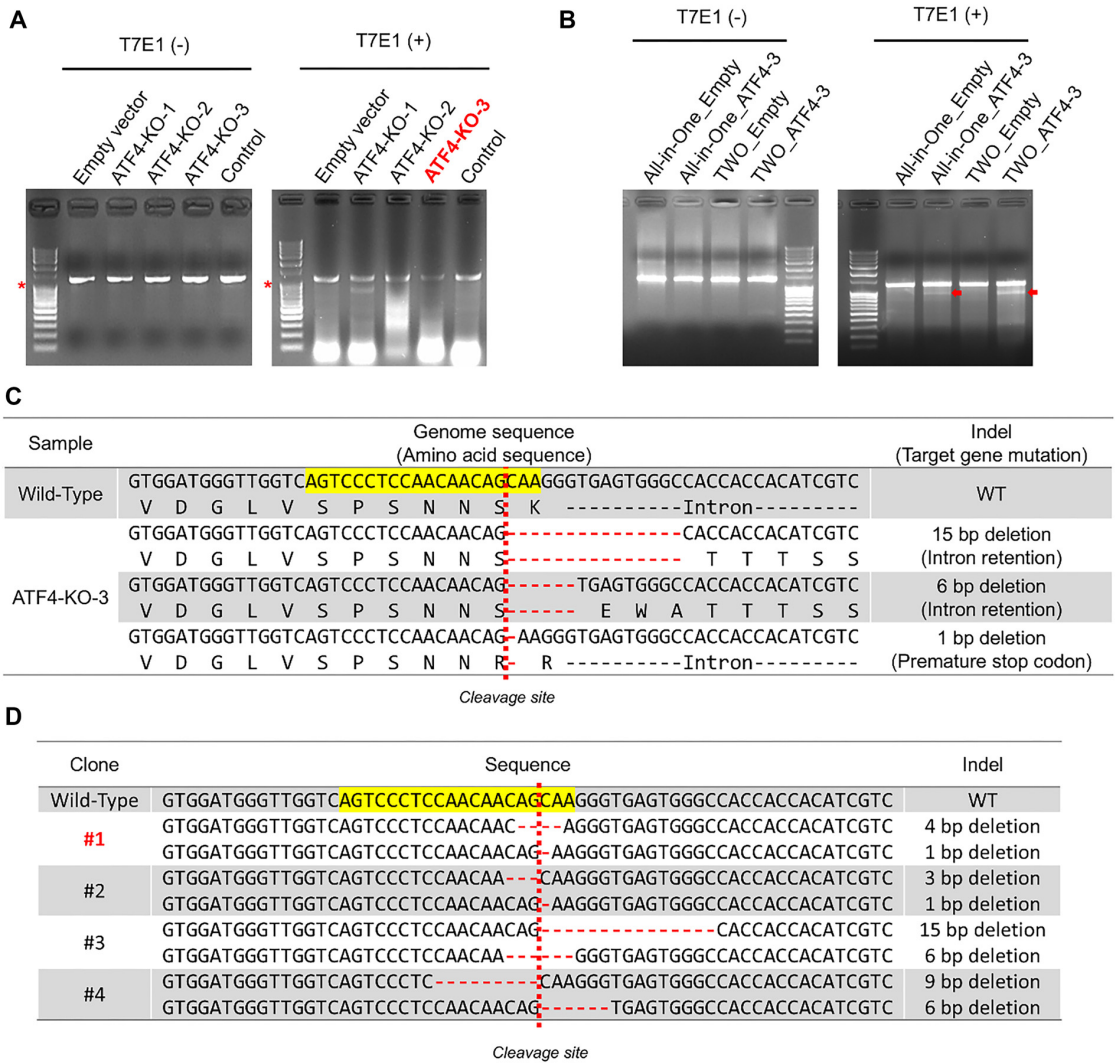
(A) T7E1 assay for sgRNA efficiency: HEK293T cells were transfected with CRISPR-Cas9 vectors expressing sgRNAs (ATF4-KO-1, −2, −3). PCR and T7E1 digestion revealed ATF4-KO-3 as the most efficient, with distinct cleavage patterns. (B) T7E1 assay for vector systems: Efficiency of all-in-one and two-vector systems was compared using ATF4-KO-3. Both systems showed effective editing, confirmed via T7E1 assay and gel analysis. (C) Gene sequencing for ATF4-KO-3: Next-generation sequencing of the *ATF4* target region identified three primary deletions (15 bp, 6 bp, 1 bp), leading to disrupted gene expression and premature stop codons (D) Single-cell clone analysis: Sequencing of single-cell clones from ATF4-KO-3 revealed distinct indel patterns, confirming successful isolation of edited clones with diverse mutations. T7E1: T7 endonuclease I; KO: Knockout; sgRNA: Single guide RNA; WT: Wild-type.

To enhance membrane protein production, sgRNAs targeting the *ATF4* gene were designed and validated. HEK293T cells were transfected with vectors expressing three sgRNAs (ATF4-KO-1, ATF4-KO-2, and ATF4-KO-3) using the all-in-one vector system, which co-expresses Cas9 and sgRNA. The efficiency of these sgRNAs was assessed using the T7E1 assay, a method that detects DNA mismatches and generates cleaved bands upon successful genome editing. Among the tested sgRNAs, ATF4-KO-3 produced the most prominent cleaved bands, highlighting its superior efficiency in editing the ATF4 gene (Figure 2A).

### Comparison of all-in-one and two-vector gene editing systems

Following the selection of ATF4-KO-3 as the most efficient sgRNA, its performance was further evaluated using two vector delivery systems: the all-in-one vector system (co-expressing Cas9 and sgRNA) and the two-vector system (expressing Cas9 and sgRNA separately). HEK293T cells were transfected with each system, and gene-editing efficiency was assessed using the T7E1 assay. The results showed that while both systems successfully induced cleaved bands indicative of editing, the two-vector system exhibited slightly higher efficiency, as evidenced by more pronounced cleaved bands on the agarose gel (Figure 2B). These findings underscore the robustness of the two-vector system for gene-editing applications.

### NGS analysis of gene editing patterns

To further investigate the gene editing patterns induced by ATF4-KO-3, NGS was performed. The sequencing results identified three major nucleotide deletions: 15 bp, 6 bp, and 1 bp. Analysis at the protein translation level revealed that the 15 bp and 6 bp deletions caused intron retention, disrupting normal gene expression. Meanwhile, the 1 bp deletion introduced a premature stop codon, effectively suppressing ATF4 protein production (Figure 2C). To validate these findings and assess the heterogeneity of gene editing, single-cell clones were generated and analyzed using NGS. Among the four clones examined, each displayed unique indel patterns, including deletions of 1 bp, 3 bp, 6 bp, 9 bp, and 15 bp. For example, clone #1 exhibited 4 bp and 1 bp deletions, while clones #2, #3, and #4 displayed various combinations of deletions (Figure 2D). These results confirmed the successful isolation of four genetically distinct clones, showcasing the diversity of gene editing outcomes.

### Establishment of ATF4-KO HEK293T single-cell clones

The ATF4-KO HEK293T cell line was generated using CRISPR-Cas9 technology. HEK293T cells were transfected with Cas9 and ATF4-KO-3 sgRNA vectors via a lentiviral delivery system. Successful transfection was verified through antibiotic selection with G418 and Zeocin, after which the selected cells were designated as ATF4-KO-3 HEK293T cells. To reduce genetic variability, single-cell clones were established using limiting dilution. Only wells containing successfully established single-cell clones were selected for further analysis. Genomic DNA from these clones was amplified to target the *ATF4* gene, and highly edited clones were identified using the T7E1 assay (data not shown). Among the selected clones, clone #1 exhibited the expected deletions in the *ATF4* gene, as confirmed by NGS (Figure 2D). This clone was subsequently designated as the ATF4-KO HEK293T cell line.

### Western blot analysis of protein expression in ATF4-KO cells

**Figure 3.**
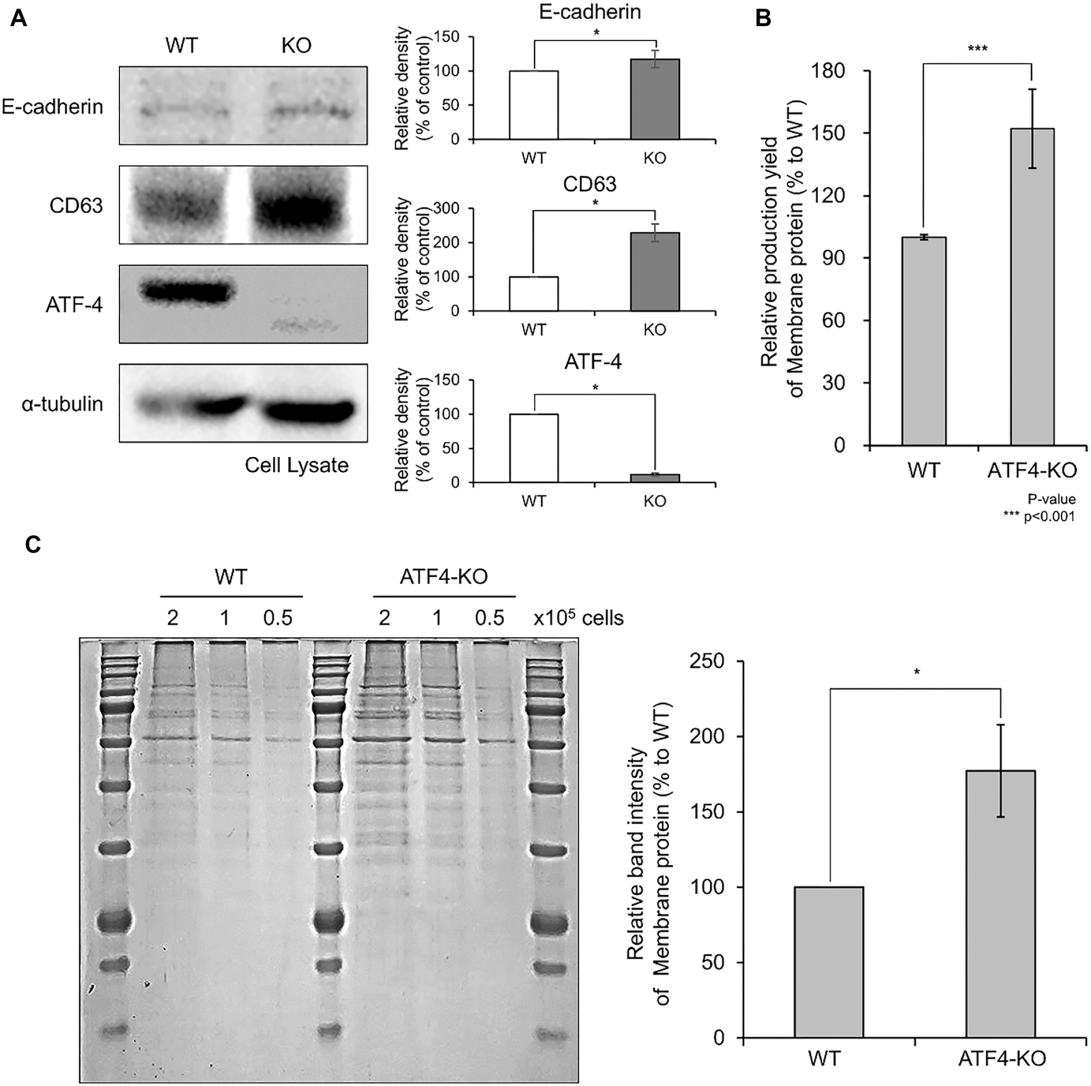
**Evaluation of membrane protein productivity in HEK293T-ATF4-KO cells.** (A) Western blot analysis to assess changes in protein expression in ATF4-KO stable cells. The expression of ATF4 was completely suppressed in ATF4-KO cells, while the expression levels of representative membrane proteins, E-cadherin and CD63, were increased. (B) Quantitative comparison of membrane protein productivity using the Bradford assay. Each sample was collected from 1 × 10^7^ cells, and membrane proteins were purified using the Membrane Extraction Kit. The concentration of purified membrane proteins was quantified using the Bradford assay, showing a 52.2 ± 19.0% increase in membrane protein productivity in ATF4-KO cells compared to WT cells (*P* < 0.001). (C) Qualitative comparison of purified membrane proteins using PAGE gel electrophoresis. Purified proteins were separated using PAGE, stained with Coomassie dye, and visualized using an LED illuminator. The intensity of protein bands was higher in ATF4-KO cells compared to WT cells. Quantitative analysis of band intensities revealed a 77.2 ± 30.6% increase in membrane protein production in ATF4-KO cells compared to WT cells. Results are presented as mean ± standard deviation from three independent experiments (*P < 0.05). WT: Wild-type; KO: Knockout.

To investigate changes in protein expression between ATF4-KO and WT cells, Western blot analysis was performed (Figure 3A). The results confirmed that ATF4 expression was completely suppressed in ATF4-KO cells. Moreover, the expression levels of the representative membrane proteins, E-cadherin and CD63, were significantly elevated in ATF4-KO cells compared to WT cells.

### Quantitative and qualitative assessment of membrane protein productivity

The membrane protein productivity of ATF4-KO cells was compared with that of WT HEK293T cells. Quantitative analysis using the Bradford assay revealed a significant 52.2 ± 19.0% increase in membrane protein production in ATF4-KO cells relative to WT cells (*P* < 0.001; Figure 3B). Furthermore, purified proteins were separated using PAGE gel electrophoresis for qualitative comparison. Consistently, ATF4-KO cells displayed more intense protein bands than WT cells, indicating higher protein yields (Figure 3C). Quantitative analysis of band intensity demonstrated a 77.2 ± 30.6% increase in membrane protein production in ATF4-KO cells compared to WT cells. These findings underscore the enhanced membrane protein productivity achieved by knocking out *ATF4* in HEK293T cells.

### Validation of membrane protein production between WT and ATF4-KO HEK293T cells

**Figure 4.**
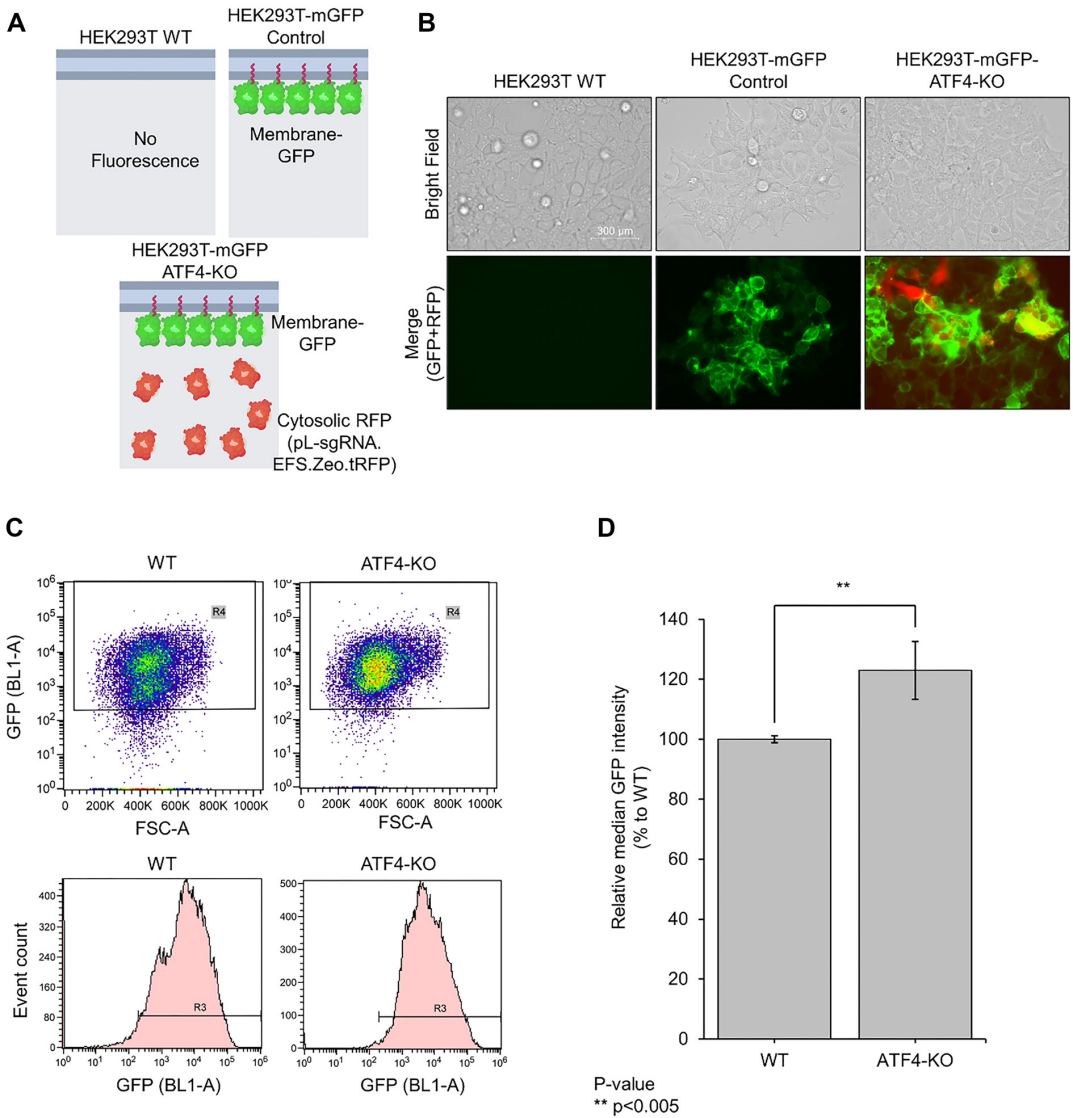
**Comparison of membrane protein productivity in the live HEK293T-ATF4-KO cells.** (A) Schematic illustration of target gene expression. To confirm the increase in membrane protein in live cells, Membrane-GFP genes were constructed and introduced into HEK293T cells to create a stable mGFP-HEK293T cell line. This stable cell line enabled the visualization of membrane-GFP expression through fluorescence microscopy. Additionally, the pL-sgRNA.EFS.Zeo.tRFP vector containing ATF4-KO-3 gRNA was delivered into these cells. The successful delivery and expression of this vector were indirectly confirmed by the presence of Cytosolic RFP, which served as a visual marker for the cells expressing the ATF4 knockout construct. (B) Representative fluorescence microscopic Images showing the distinct expression patterns of the cell lines. HEK293T WT cells displayed no fluorescence, confirming the absence of GFP and RFP signals. HEK293T-mGFP cells exhibited GFP expression, visible in the GFP channel, and HEK293T-mGFP-ATF4-KO cells showed fluorescence in both GFP and RFP channels, confirming the successful integration and expression of both GFP and RFP in the edited cell line. (C) Flow cytometry showing the changes in GFP intensity in HEK293T-mGFP control and HEK293T-mGFP-ATF4-KO cells. Analysis was conducted using dot plot (top) and histogram (bottom), respectively. Two groups were confirmed for HEK293T-mGFP control, a low GFP intensity group (10^3^) and a high GFP intensity group (10^4^), but ATF4-KO cells were confirmed to be mostly in the high GFP intensity group (10^4^). (D) Median GFP intensity comparison. The difference in GFP intensity between control cells and ATF4-KO cells was compared and analyzed through the median value of GFP intensity. A 22.9 ± 9.7% increase in GFP intensity was confirmed in ATF4-KO cells compared to WT cells (*P* < 0.005). Values are presented as mean ± standard deviation of three independent experiments. *P < 0.05. KO: Knockout; mGFP: Membrane-bound form of green fluorescent protein; WT: Wild-type.

To confirm increased membrane protein expression in live cells, mGFP genes were constructed and introduced into HEK293T cells to establish a stable mGFP-HEK293T cell line (Figure 4A). The pL-sgRNA.EFS.Zeo.tRFP vector, containing ATF4-KO-3 gRNA, was subsequently delivered into these cells, with its expression indirectly confirmed by the presence of cytosolic RFP. Fluorescence microscopy revealed distinct expression patterns in the cell lines (Figure 4B). HEK293T WT cells showed no fluorescence, confirming the absence of GFP and RFP signals. In contrast, HEK293T-mGFP cells exhibited GFP fluorescence in the GFP channel, while HEK293T-mGFP-ATF4-KO cells showed fluorescence in both GFP and RFP channels, validating the successful integration and expression of both proteins in the edited cell line. Flow cytometry was then used to measure GFP intensity in the stable cell lines expressing mGFP, sgRNA, and Cas9 after two weeks of maintenance (Figure 4C). Dot plots and histograms revealed two distinct GFP intensity populations in WT cells: a low-intensity group (10^3^) and a high-intensity group (10^4^). In contrast, the ATF4-KO stable cell line predominantly exhibited only the high GFP intensity population (10^4^). Comparative analysis of median GFP intensity demonstrated a 22.9 ± 9.7% increase in GFP intensity in the ATF4-KO cell line compared to the WT line (Figure 4D). These results indicate a significant enhancement in membrane protein production in ATF4-KO cells.

**Figure 5.**
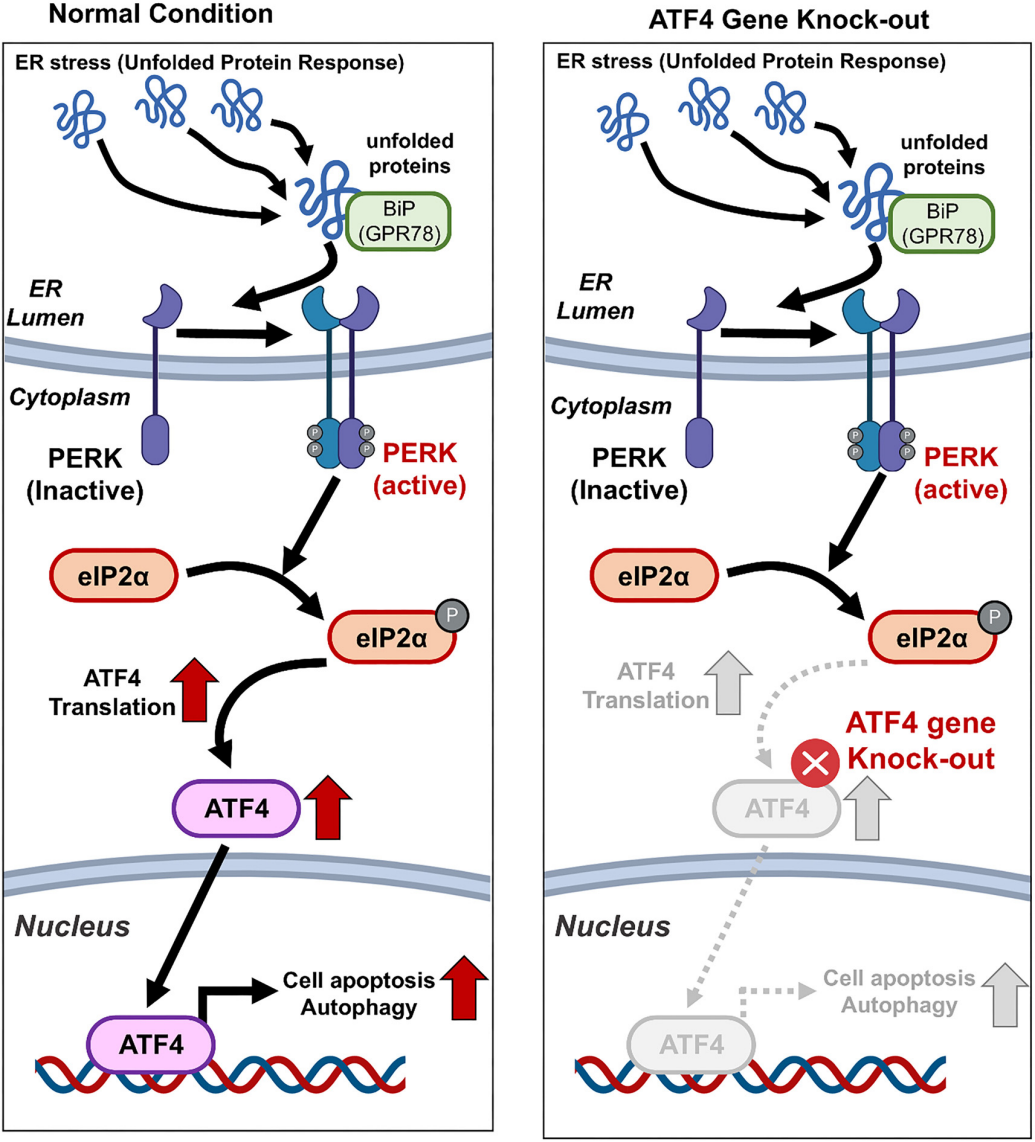
**Schematic diagram of PERK signaling pathway involved in the UPR.** The BiP (GPR78) proteins that first recognize ER stress during the UPR process. Unfolded protein-binding BiP was recognized by PERK is transmitted downstream. PERK activates the down-stream target gene, eIF2α, through phosphorylation. Afterwards, eIF2α induces the transcription and translation of ATF4 while suppressing protein translation. ATF4, with increased expression, is involved in programmed cell death and cell survival, respectively, depending on its binding partner. In this study, we directly knocked out ATF4 and suppressed the transcriptional activity caused by ATF4, thereby leading to increased productivity of membrane proteins. UPR: Unfolded protein response; PERK: Protein kinase RNA-like endoplasmic reticulum kinase; ER: Endoplasmic reticulum.

## Discussion

In this study, membrane protein production in HEK293T cells was enhanced by knocking out the *ATF4* gene using CRISPR-Cas9 technology. The experimental approach involved optimizing sgRNA efficiency, validating gene editing via T7E1 assays and NGS, and comparing the performance of all-in-one vs two-vector delivery systems. The most efficient sgRNA, ATF4-KO-3, was identified and used to generate stable ATF4-KO HEK293T cell lines. These cell lines exhibited a significant increase in membrane protein production compared to WT cells. Quantitative analysis revealed a 52.2 ± 19.0% increase in membrane protein production, while PAGE gel electrophoresis showed a 77.2 ± 30.6% increase in protein band intensity. Additional validation using Membrane-GFP expression and flow cytometry demonstrated a 22.9 ± 9.7% enhancement in GFP intensity in ATF4-KO cells relative to WT cells. These findings strongly support the notion that ATF4-KO improves membrane protein production, underscoring its potential for biotechnological applications. The results suggest that knocking out *ATF4* can increase membrane protein production by mitigating ER stress. This is because the *ATF4* gene plays a critical role in the unfolded protein response (UPR), a cellular mechanism that responds to the accumulation of misfolded or unfolded proteins in the ER [13–15]. The UPR is primarily mediated by three key ER stress sensors: ATF6, PERK, and IRE1, which detect the buildup of misfolded proteins and activate downstream signaling pathways [16–18]. During ER stress, BiP (GRP78), a chaperone protein, dissociates from these stress sensors—ATF6, PERK, and IRE1—triggering their activation [14, 19]. Among these pathways, PERK phosphorylates eIF2α, leading to the activation of *ATF4* [20, 21]. Once activated, ATF4 regulates genes involved in cell survival or apoptosis, depending on its binding partners. However, the PERK-eIF2α-ATF4 pathway is predominantly associated with programmed cell death in response to ER stress. By knocking out *ATF4*, cells can bypass apoptosis caused by ER stress [13, 19, 20]. This enables them to survive longer and allocate more resources toward membrane proliferation and protein synthesis. As a result, reducing ER stress via *ATF4* KO facilitates higher yields of membrane proteins. The findings of this study highlight the utility of *ATF4* KO as a strategy for optimizing membrane protein production in HEK293T cells, offering new opportunities for biotechnological advancements Figure 5.

In this study, a novel method to enhance membrane protein production in HEK293T cells was developed. The resulting proteins can be classified and utilized across various bioindustries. For example, lipid nanoparticles—composed of lipid components—could benefit from conjugation with membrane proteins that possess suitable isoelectric points, improving their biocompatibility and binding affinity with cell membranes. Compared to conventional strategies, such as optimizing culture conditions, transient transfection, or stable cell line development, the *ATF4* KO approach offers a unique advantage by directly targeting cellular stress response pathways [3, 6]. For example, while methods like chaperone overexpression or media optimization primarily improve protein folding and trafficking, *ATF4* KO mitigates ER stress at its source by suppressing the PERK-eIF2α-ATF4 pathway [7, 12]. This not only enhances cell survival under stress conditions but also redirects cellular resources toward protein synthesis. Previous studies have used genetic modifications, such as deleting lipid metabolism regulators like PAH1 in yeast, to promote ER membrane expansion and boost protein production [22, 23]. However, these approaches typically rely on non-human cell lines, which limits their utility for therapeutic protein production. In contrast, using HEK293T cells, as demonstrated here, ensures human-like post-translational modifications that are critical for clinical-grade membrane protein production [1, 17]. Thus, our findings establish ATF4 KO as a scalable and effective strategy that complements existing methods for improving membrane protein yields. Despite these promising results, several limitations must be addressed in future research. First, the efficiency of the CRISPR-Cas9 system in generating stable KO cell lines may vary, resulting in heterogeneous populations of edited cells [24]. This heterogeneity could affect the consistency and reproducibility of membrane protein production. To address this, single-cell cloning and rigorous validation of KO cells are necessary to ensure uniform and stable gene editing outcomes [25, 26]. Second, while this study demonstrated increased membrane protein production through *ATF4* KO, the broader implications for cellular metabolism and stress responses were not extensively explored. Comprehensive analyses of potential off-target effects and the overall cellular health post-ATF4 KO are needed to ensure that other critical cellular functions are not inadvertently impaired. Additionally, the scalability of this enhanced membrane protein production in HEK293T cells for industrial applications remains unvalidated. Future studies should focus on optimizing bioreactor conditions and scaling up the production process while maintaining high yields and protein functionality. Finally, although flow cytometry and fluorescence microscopy provided valuable insights into protein expression levels, more quantitative and qualitative assays—such as mass spectrometry and functional tests—are required to gain a deeper understanding of the structural and functional integrity of the produced membrane proteins.

## Conclusion

In conclusion, the KO of the *ATF4* gene in HEK293T cells significantly enhances membrane protein production. Quantitative analysis via the Bradford assay revealed a 52.2 ± 19.0% increase in membrane protein yield, while PAGE gel electrophoresis demonstrated a 77.2 ± 30.6% rise in the band intensity of purified membrane proteins. Western blot analysis further confirmed the suppression of *ATF4* expression and the corresponding increase in E-cadherin and CD63 levels, validating the enhanced membrane protein production. These findings highlight the pivotal role of *ATF4* in protein regulation and underscore the utility of CRISPR-Cas9 technology for optimizing cellular functions. This approach provides a powerful tool for boosting protein yields, with significant implications for drug development, structural biology, and protein engineering.

## Data Availability

The datasets generated and/or analyzed during the current study are available from the corresponding author upon reasonable request.
